# The Treatment of Internal Hæmorrhage

**Published:** 1892-09-17

**Authors:** 


### SURGERY.

### THE TREATMENT OF INTERNAL HÆMORRHAGE.

Hæmorrhage from the lungs, stomach, or intestines is seldom to such an extent as to prove immediately fatal on its first occurrence; were it so, physicians would have few opportunities of making use of those drugs or applying those physical means usually adopted to arrest or restrain this symptom. The treatment is in each case the same, with certain but most important differences, and, after detailing the drugs and means employed in general, a few words will suffice to point out what medicine has been found under certain circumstances to act most rapidly and most efficaciously in particular diseases.

We have seldom the chance of making use of any prophylactic measures, as the hæmorrhage, perhaps not unexpected as a complication of the disease, occurs at times when we least look for it; though, in a few diseases, by careful dieting or by reducing the blood pressure, we may ward off a threatening attack. For instance, when the diagnosis of gastric ulcer has been made, or we suspect that the patient is suffering from malignant disease of the stomach, a free action of the bowels may prevent a possible attack, and in no case is the beneficial use of free purgation better seen than in cases of hepatic cirrhosis with venous congestion of the mesenteric veins. In consumption we have little warning in the majority of cases that hæmorrhage is about to occur, and the reduction of the patient's strength by the use of drastic purgatives would not be treatment that we should recommend. Hæmorrhage from tubercular ulcers of the intestine seldom occurs, but one of the gravest complications of typhoid fever is the ulceration of the walls of the arteries at the base of the ulcer. No one would think of purgation under these conditions, though many physicians advise the use of a single purgative at the early part of the disease. It will be found, however, that the use of enemata alone is attended with far less danger than that of purgatives. It has been suggested that in those cases of typhoid in which constipation is present that the injection of water, often with turpentine, causes the hardened masses of fœces so frequently seen in typhoid fever to scrape the surface of the ulcers, and so to cause the very complication which it is our desire to avoid, but the risk in giving enemata and the evacuation of the bowel by this means is less than an expectant policy would offer.

Constitutional treatment in such diseases as hœmophilia, purpura, and the like, may ward off attacks, but both this and the use of hæmostatic drugs is often followed with indifferent success.

Passing on then to the use of hæmostatic drugs and other means of arresting hæmorrhage, we shall notice first those methods employed to reduce the blood-pressure by encouraging the cardiac depression by dilatation of the vascular area, and those astringent drugs used to contract the open mouths of bleeding vessels.

In encouraging cardiac depression we must avoid weakening the power of the heart, a condition not produced by such drugs as opium, morphia, or turpentine, or means such as mental and bodily rest. Purgatives will reduce the blood-pressure by withdrawal of a large quantity of the fluid constituents of the blood, and are much used in pulmonary and gastric hæmorrhage, but they must be used with caution. In cases of cerebral hæmorrhage a smart purgative often does considerable good, croton oil, calomel, and other rapidly-acting drugs being used.

Where the bleeding point is in a position to be acted on directly by the drugs, nitrate of silver, acetate of lead, preparations of ergot and local cold are employed. In speaking of these a few words must be added on the use of ergot. This drug, from the action it possesses on the uterus, causing its contraction and arresting hæmorrhage from uterine sinuses, is often employed to control bleeding from other organs; and it must be added that a certain amount of good seems to follow its use, but we must remember that the conditions under which it acts in other parts than the uterus are entirely different. The uterine sinuses have no muscular construction of their own, but are closed by the contraction of the uterine muscular fibres. This contraction is much assisted by the use of ergot, which appears to act on the nervous mechanism of the uterus far more energetically than on other involuntary muscle. Ergot, it is true, acts to some extent on the muscular tissue of the arteries, causing contraction, and at the same time increasing the arterial tension, but diminishing the flow of blood, but its action is insignificant compared with what it has on the uterus, and the frequent failure to arrest hæmorrhage with the use of this drug is not to be wondered at when we consider these facts. The application of cold by an ice bag is often of great value.

Hæmostatic or styptic drugs act in three ways: (1) on the blood; (2) by causing contraction of the broken vessel; (3) by constricting the surrounding parts and so causing pressure on the vessels, but they may also act in two or even in all three of these ways. The changes they produce in the blood consist in hastening its coagulation and so in assisting the formation of a clot at the open mouth of the vessel. The drugs employed to produce this effect are persalts of iron, sulphate of copper, acetate of lead, dilute mineral acids, alum, catechu, kino and its allies, such as rhatany, galls, &c.; and contraction of the vessels is produced by acetate of lead, nitrate of silver, ergot, and the application of cold; and we have tannin, catechu, salts of lead, silver, copper, and zinc, and the persalts of iron as constricting drugs.

Which of these drugs should be used under particular circumstances must now occupy our attention for a short time, for in certain diseases one drug seems to act in a much more efficacious manner than when used for the treatment of hæmorrhage in another case.

In pulmonary hæmorrhage, attempts must be made to keep the patient as quiet as is possible under the circumstances. Examination of the chest should not be conducted for fear of disturbing any forming clot on a vessel, an accident which has happened, and one which the medical adviser would rather avoid if possible. The application of ice to the supposed seat of hæmorrhage is advisable, though our knowledge of its locality is often small. A hypodermic injection of morphia, or laudanum by the mouth in full doses should be given immediately with a ʒi of turpentine, to be repeated in smaller doses should the hæmorrhage continue. Few drugs will be found to be more useful in pulmonary hæmorrhage than turpentine, which causes a decided fall in blood-pressure, besides having a sedative effect on the central and spinal centres. Its frequent use is, however, attended in some cases with irritation and congestion of the urinary organs, leading at times to hæmaturia, painful micturition, and even suppression of urine.

In gastric hæmorrhage, whether from an ulcer or from malignant growth, the use of cold applications, morphia, and tannic acid is often attended with success. The latter, though it does not actually contract the vessels but rather dilates them, causes a marked constricting influence on the tissues. Persalts of iron, such as the perchloride, also have a strong effect, and dieting of the patient must occupy our attention, all food being given by the rectum if the symptoms are alarming. Gastric sedatives must also be combined with astringent drugs, the carbonate and nitrate of bismuth acting most efficaciously, and perhaps assisting coagulation from the presence of the small solid particles. Opium or morphia must also be given freely, and though some advise that purgatives should be given to diminish the blood supply to the stomach, perfect rest will be found to be of more value. Hamamelis is one of the newest drugs recommended for hæmmorhage, and in the hands of some has proved to be very useful; it is given as the tincture.

As soon as hæmorrhage is discovered in enteric fever, the most energetic means must be taken to arrest it, for there is no certain means by which we can tell how much blood is being poured out into the intestines, and the patient may bleed to death without any blood being passed by the rectum. Opium or morphia in full doses, the former by the mouth or with an enema of starch, the latter hypodermically, tannic or gallic acid, and cold applications on the abdomen are the routine treatment. Turpentine is often given, but must be guarded with opium, as it otherwise increases peristaltic movements, and may assist the hæmmorrhage rather than arrest it. Acetate of lead with opium is often given, and with marked success, but we should expect more satisfactory results from hæmostatic drugs than from styptic or astringent ones.

Ice water injections into the rectum have also been used, but are not of any special value. What is required is a hæmostatic drug which will act as rapidly as apomorphine does on the vomiting centre.

